# Continuous Estimation of Cardiac Output in Critical Care: A Noninvasive Method Based on Pulse Wave Transit Time Compared with Transpulmonary Thermodilution

**DOI:** 10.1155/2020/8956372

**Published:** 2020-07-20

**Authors:** Ulrike Ehlers, Rolf Erlebach, Giovanna Brandi, Federica Stretti, Richard Valek, Stephanie Klinzing, Reto Schuepbach

**Affiliations:** ^1^Institute of Intensive Care, University Hospital of Zurich, Raemistasse 100 CH-8091, Zurich, Switzerland; ^2^Intensive Care Unit, Kantonsspital Glarus, Burgstrasse 99, CH-8750, Glarus, Switzerland

## Abstract

**Purpose:**

Estimation of cardiac output (CO) and evaluation of change in CO as a result of therapeutic interventions are essential in critical care medicine. Whether noninvasive tools estimating CO, such as continuous cardiac output (esCCOTM) methods, are sufficiently accurate and precise to guide therapy needs further evaluation. We compared esCCOTM with an established method, namely, transpulmonary thermodilution (TPTD). *Patients and Methods*. In a single center mixed ICU, esCCOTM was compared with the TPTD method in 38 patients. The primary endpoint was accuracy and precision. The cardiac output was assessed by two investigators at baseline and after eight hours.

**Results:**

In 38 critically ill patients, the two methods correlated significantly (*r* = 0.742). The Bland–Altman analysis showed a bias of 1.6 l/min with limits of agreement of −1.76 l/min and +4.98 l/min. The percentage error for CO_esCCO_ was 47%. The correlation of trends in cardiac output after eight hours was significant (*r* = 0.442), with a concordance of 74%. The performance of CO_esCCO_ could not be linked to the patient's condition.

**Conclusion:**

The accuracy and precision of the esCCOTM method were not clinically acceptable for our critical patients. EsCCOTM also failed to reliably detect changes in cardiac output.

## 1. Introduction

The hemodynamic monitoring of critical care patients with hypovolemia, myocardial dysfunction, or alterations in vascular tone is essential in assessing their condition and tailoring volume and the vasoactive therapy [1]. Continuous bedside monitoring of cardiac output (CO) allows the recording of changes in cardiac function and evaluation of responses to the therapy, such as volume challenges or administration of medication [1]. While several techniques for assessing hemodynamic function already exist [2], noninvasive or minimally invasive methods are emerging. The pulmonary artery catheter (PAC) is considered the gold standard method for monitoring cardiac output, but it is associated with the risks of central venous catheterization (e.g., dysrhythmias) and catheter residence (e.g., venous thrombosis, pulmonary embolism and infarction, pulmonary artery rupture, and sepsis [3]) and fails to record CO stroke by stroke. The widespread use of PAC decreased after its benefit was questioned in a systematic review [4]. Potentially less-invasive devices, such as PiCCOTM (Pulsion Medical Systems, Germany), have been considered preferable by some institutions [5]. The PiCCOTM is an intermittent transpulmonary thermodilution (TPTD) based on periodical calibration and the arterial pulse contour analysis for continuous, stroke by stroke estimation of CO. Acceptable agreement on the estimation of CO by TPTD and PAC was found in critically ill patients [6] and between the arterial pulse contour analysis and TPTD in hemodynamically unstable patients [7]. However, as PiCCOTM requires the placement of a central venous catheter and a femoral artery catheter, complications of cannulation have to be considered [8, 9].

The survival benefit due to CO monitoring is still uncertain [5, 10], and efforts to develop minimally invasive devices allowing estimation of CO are ongoing. One of these is VismoTM (Nihon Kohden, Japan), a device that continuously estimates the CO (esCCOTM) based on the pulse wave transit time (PWTT) method. The PWTT is in theory inversely correlated with the left ventricular stroke volume [11]. It measures the time between the *R*-Wave in the electrocardiogram and the appearance of the pulse wave by pulse oximetry on a finger. The validity of the technique needs to be assessed in an ICU population, and its impact on outcome, if used to guide therapeutic decisions, is uncertain [2].

As a noninvasive method allowing continuous estimation of CO, the esCCOTM technique represents an alternative to more invasive monitoring methods. However, whether it shows acceptable accuracy and precision and whether it can properly detect changes in CO in ICU patients remains to be established. This study therefore compares esCCOTM estimated by VismoTM to the CO determined by PiCCOTM and tests whether the two devices show similar efficacy in detecting a change in CO in a mixed ICU population.

## 2. Material and Methods

### 2.1. Patients

Patients hospitalized from January to December 2016 in a critical care unit at the University Hospital Zurich with invasive hemodynamic monitoring in place were eligible for this study. The study protocol was approved by the Ethics Committee Zurich (KEK-ZH-No. 2016-0666) and was designed in accordance with the Declaration of Helsinki. Patients were eligible if between 18 and 100 years of age and if they already had a PiCCOTM system and a central venous catheter in place. Noninvasive blood pressure measurement had to be taken on one arm, and ECG electrodes and finger pulse oximetry needed to be attached. Patients with a diagnosis of aortic aneurysm, atrial fibrillation, or ventricular arrhythmia were excluded, as were pregnant women. In addition, patients were excluded if scheduled for mobilization out of bed during the study period or if allergic to any components of the equipment used for the study. Written informed consent was obtained from all patients or their next of kin.

### 2.2. Study Procedures

Patients included were assigned to one of three predefined subgroups (“sepsis,” “polytrauma,” or “nonsepsis/nonpolytrauma”) according to their diagnosis. Using the electronic patient file (KISIM, Cistec, Zurich, Switzerland), concomitant diagnoses and the simplified acute physiology score 2 (SAPS II) were registered. The SAPS II score is a scoring system for the predicted average ICU mortality and is regularly calculated in the first 24 hours of ICU admission [12]. For study purposes, the SAPS II score was calculated again at the time of first measurement. The variables heart rate, arterial blood pressure (systolic, diastolic, and mean), respiratory rate, and saturation of peripheral oxygen (SpO2) were assessed via the PiCCOTM monitor. At baseline, the esCCOTM system was applied (three ECG electrodes, one blood pressure cuff, and a one finger pulse oximeter). The PiCCOTM device was recalibrated according to manufacturer's standard. For calibration of the esCCOTM, the investigator entered gender, age, height, and weight of the patient and ran a noninvasive blood pressure measurement. CO was then documented from both the PiCCOTM and the VismoTM (M_baseline #1_). This initial procedure was repeated by a second investigator (M_baseline #2_). At least one of the two investigators was a senior ICU physician. After 8 hours, CO was obtained from both devices (M_8 h_). After M_8 h_, the VismoTM device was removed, and the study phase was finished.

### 2.3. Statistical Analysis

A sample size of 40 patients was chosen to limit the total width of the 95% confidence interval of the percentage error to 15%, calculated on a hypothetical percentage error of 30% and mean cardiac output of 5 l/min [13].

For descriptive statistics, categorical variables were expressed as absolute numbers with percentages, normally distributed quantitative variables as mean ± standard deviation (SD) and nonnormally distributed variables as median with interquartile range (IQR). For the data analysis, the mean of *M*_baseline #1_ and *M*_baseline #2_ (*M*_mean baseline_) was used to minimize interobserver variability. Linear correlation between CO_esCCO_ and CO_TPTD_ was assessed with the Pearson product-moment correlation coefficient (*r*). Agreement between methods of measurement was calculated using the Bland–Altman analysis. Accuracy is represented by the mean difference between CO_esCCO_ and CO_TPTD_ (bias). Precision is represented by the standard deviation (SD) of difference in CO in one device at one time point. Limits of agreement (LoA) are calculated as bias +1.96 × SD (upper LoA) and bias −1.96 × SD (lower LoA). The percentage error (1.96 × SD of difference in CO/mean CO of the two methods × 100%) was considered clinically acceptable if it was lower than 30% [14]. 95% confidence intervals (95% CI) were calculated for the Bland–Altman analysis as bias ± 1.96 × SD/$\sqrt{n}$ and upper and lower LoA ± 1.96 × $\sqrt{3}$ × SD/$\sqrt{n}$, where *n* is the sample size. 95% CI for the percentage error was calculated as (upper 95% CI limit of the upper LoA − bias)/mean CO × 100% and (lower 95% CI limit of the upper LoA −bias)/mean CO × 100% [13]. Interobserver variability between *M*_baseline #1_ and *M*_baseline #2_ was evaluated by the Bland–Altman analysis.

Trending ability was assessed between cardiac output after calibration (*M*_mean__baseline_) and after 8 hours (*M*_8 h_) using a four-quadrant plot and the Pearson product-moment correlation coefficient (*r*). Concordance rate was calculated as percentage of all cases when the trends (ΔCO_esCCO_ and ΔCO_TPTD_) were in the same direction. A concordance rate of 90–95% was considered a reliable trending ability [15].

All tests were two-sided, and a *p* value <0.05 was considered statistically significant. The statistical analysis was performed using SPSS Statistics version 23 (IBM Corporation, Armonk, NY, USA). Raw data are provided (supplementary data (available here)).

## 3. Results

### 3.1. Patients

From January 1^st^, 2016 to December 31^st^, 2016, a total of 1,689 patients were treated in our ICUs. Hemodynamic monitoring with PiCCOTM was implemented in 128 patients. Of those, 40 patients fulfilled the inclusion criteria and consented to be enrolled in the study. One patient was excluded because of consent withdrawal before the first measurement. In another patient, the PiCCOTM measurements failed. Consequently, the data analysis was performed for 38 patients. Data for the 8-hour follow-up measurement were available from 34 patients. Missing the follow-up resulted from death in one patient, measurement failure of the VismoTM device in one patient, and in two patients, the necessary removal of the PiCCOTM-line. Of the 38 patients enrolled, 26 (68%) were male. The median age was 62 (IQR: 50–72) years. Indications for hemodynamic monitoring were sepsis in 13 (34%) cases, polytrauma in 6 (16%) cases, sepsis and polytrauma in 1 (3%) case, and neither sepsis nor polytrauma in 18 (47%) cases. Patients' characteristics are provided in Table 1.

### 3.2. Agreement between the Two Methods

**Figure 1:**
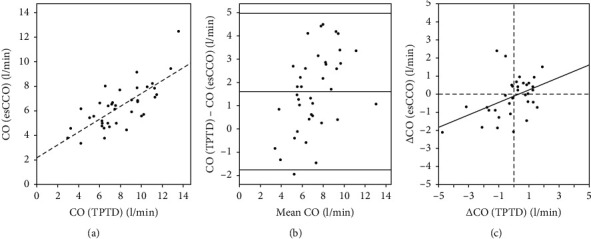
Comparison between CO_TPTD_ and CO_esCCO_. (a) Correlation between CO_TPTD_ and CO_esCCO_ estimates at baseline. Each dot represents one patient. Correlation between the two estimates was significant (*r* = 0.742, *p* < 0.001). The regression coefficient was 0.52, and the intercept was 2.21 l/min (CO_esCCO_ = 0.52 × CO_TPTD_ + 2.21 l/min) indicating that at low CO, the esCCO was overestimated and at high cardiac output, underestimated CO as compared to the TPTD method. (b) The Bland–Altman plot of CO_TPTD_ and CO_esCCO,_ Each dot represents a pair of simultaneous cardiac output measurements by esCCO and TPTD of the same patient. The midhorizontal line marks the average difference between CO_TPTD_ and CO_esCCO_ (bias; 1.61 l/min). The upper and lower horizontal lines represent the 95% confidence interval of the difference between CO_TPTD_ and CO_esCCO_ (limits of agreement; −1.76 and +4.98 l/min). (c) The four quadrant plot of the correlation between ΔCO_esCCO_ and ΔCO_TPTD_. Each dot represents the change of cardiac output over an 8-hour period (*M*_8 h_ − *M*_mean baseline_) assessed by transpulmonary thermodilution (TPTD) and continuous cardiac output (esCCO). The regression fitted (ΔCO_esCCO_ = 0.35 × ΔCO_TPTD_ − 0.09 l/min) supported that a change in cardiac output overtime was underestimated by the esCCO as compared to the TPTD method.

To test for precision, CO measured by PiCCOTM (CO_TPTD_) was compared with CO estimated by VismoTM (CO_esCCO_). The average CO measurements at baseline (*M*_mean baseline_) by CO_TPTD_ correlated significantly with CO_esCCO_ estimates (*r* = 0.742, *p* < 0.001, Figure 1(a)). The bias on an average was 1.6 l/min (95% CI of 1.1 l/min to 2.2 l/min) with a lower LoA of −1.76 l/min (95% CI of −2.71 l/min to −0.81 l/min) and an upper LoA of +4.98 l/min (95% CI of +4.03 l/min to +5.92 l/min) (Figure 1(b)). The percentage error was 47% (95% CI of 34% to 60%).

To test for interobserver variability, a patient's CO assessed by a senior ICU physician was compared with that assessed by another physician. Mean bias between observers was −0.3 l/min for CO_TPTD_ and −0.1 l/min for CO_esCCO_. Corresponding limits of agreement were −1.9 l/min and +1.3 l/min and −1.7 l/min and +1.5 l/min, respectively.

### 3.3. Trending Ability

To test whether methods reported a change in CO similarly, CO assessed at baseline (*M*_mean baseline_) was compared with CO after 8 hours (*M*_8 h_). Correlation between 8-hour trends of CO_esCCO_ (ΔCO_esCCO_) and CO_TPTD_ (ΔCO_TPTD_) was significant (*r* = 0.442, *p*=0.009; Figure 1(c)). In all, 19.5% of changes in CO measured with esCCOTM accounted for changes in CO measured with TPTD (*r*^2^ = 19.5%). The concordance rate of trends was 74%.

### 3.4. Performance in Patients with Sepsis, Polytrauma, and Other Conditions

Comparisons between techniques were planned for 3 predefined subgroups: patients with sepsis, patients with polytrauma, and patients with neither sepsis nor polytrauma.

**Figure 2:**
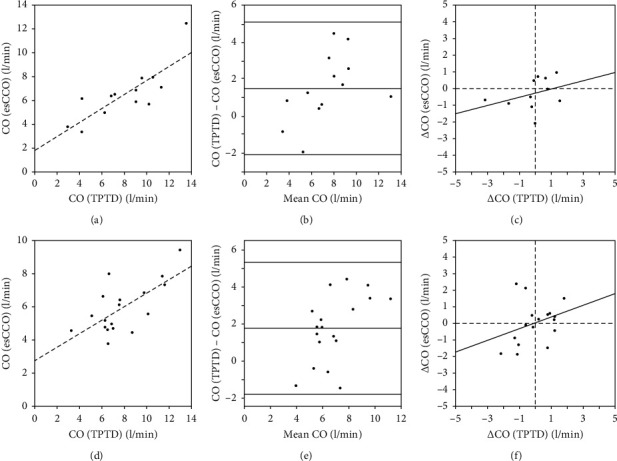
Comparison between CO_TPTD_ and CO_esCCO_ in patients with sepsis (a–c) and patients with neither sepsis nor trauma (d–f). (a) In patients with sepsis, correlation between COTPTD and COesCCO estimates at baseline was significant (*r* = 0.813, *p*=0.001), regression coefficient was 0.59, and the intercept was 1.82 l/min (COesCCO = 0.59 × COTPTD + 1.82 l/min); (d) in patients with neither sepsis nor trauma, correlation was significant (*r* = 0.669, *p*=0.002), regression coefficient was 0.41, and the intercept was 2.75 l/min (COesCCO = 0.41 × COTPTD + 2.75 l/min). In patients with sepsis (b) comparing COTPTD and COesCCO yielded a bias of 1.52 l/min and limits of agreement of −2.08 and +5.12 l/min; in patients with neither sepsis nor trauma (e), bias was 1.77 l/min and limits of agreement of −1.81 and +5.34 l/min. Estimates of the change in CO over the first 8 h (*M*8 h – *M*mean baseline) was analyzed by four quadrant plots with regressions fitted. In patients with sepsis (c), ΔCOesCCO = 0.24 × ΔCOTPTD – 0.27 l/min, and for patients with neither sepsis nor trauma (f), ΔCOesCCO = 0.35 × ΔCOTPTD + 0.02 l/min.

Among 18 patients with neither sepsis nor polytrauma, CO_esCCO_ and CO_TPTD_ correlated significantly (*r* = 0.669, *p*=0.002) with a percentage error of 53% (Figure 2(d)). Among the subgroup of patients with sepsis (*n* = 13), the two CO estimates correlated significantly (*r* = 0.813, 0.001) with a percentage error of 49% (Figure 2(a)), whereas the 6 patients with polytrauma were not analyzed as planned because of the small group size. The one patient with polytrauma and sepsis was excluded from analyzes. CO_esCCO_ as compared to CO_TPTD_ underestimated CO in all subgroups (Figures 2(b) and 2(e)), and analyzing trends in subgroups was not significant (Figures 2(c) and 2(f)).

## 4. Discussion

This prospective controlled study compares noninvasive esCCOTM estimates with CO assessed by transpulmonary thermodilution. In our mixed ICU population, the two techniques had poor agreement with a bias of 1.6 l/min and broad limits of agreement, a lower LoA of −1.76 l/min, and an upper LoA of +4.98 l/min. The percentage error of esCCOTM was 47% and thus not acceptable for clinical use. We found that esCCOTM fails to detect changes in cardiac output, as the concordance rate of trends was as low as 74%. Similarly, changes in cardiac output estimated by TPTD only predicted 19.5% of the changes observed by esCCOTM. Dividing the study population into predefined subgroups according to underlying condition identified patients with sepsis to be best suited for CO estimation by esCCOTM.

To our knowledge, this is the first study, apart from studies of patients undergoing anesthesia, to compare the pulse-wave transition time method esCCOTM with TPTD in a mixed ICU population. Reliable noninvasive tools for monitoring cardiac function are highly desirable [2]. However, techniques currently available and evaluated for clinical practice allow intermittent measurement of cardiac output (e.g., echocardiography) or need invasive calibration (e.g., the pulse-contour analysis with transpulmonary thermodilution). It can be assumed that noninvasive methods offer several potential advantages such as the following: use without extensive training, screening of patients in need of invasive monitoring, and immediate benefit from the volume therapy based on cardiac output, use in primary and secondary transport, and reduction of complications associated with invasive procedures and catheters. Noninvasive devices are, in theory, easy and fast to install. In practice, our investigators sometimes needed up to two hours to obtain the first measurement, because the VismoTM device failed to calculate the cardiac output. As the esCCOTM method appears to offer all these advantages, our goal was to investigate its interchangeability with our current standard hemodynamic monitor PiCCOTM.

Our first goal was to analyze the accuracy and precision of esCCOTM compared with TPTD in critically ill patients. Our data showed a strong correlation between CO_TPTD_ and CO_esCCO_ (*r* = 0.742), in concordance with various previous studies that compared CO_esCCO_ with CO from pulmonary artery catheter thermodilution [16–18] and transthoracic echocardiography [19, 20]. However, the bias of esCCOTM in the Bland–Altman analysis (Figure 1(b)) was high (1.6 l/min) and its range of agreement was broad (−1.76 l/min; +4.98 l/min), indicating low accuracy and low precision for this method in our study population, and thus rendering the device clinically unacceptable for ICU patients. Yamada et al. [16], Terada et al. [17], and Mansencal et al. [21] reported the accuracy and precision superior to our findings. As the study of Terada et al. [17] included intraoperative patients only, their findings may not be generalizable to critical care patients. Another study used cardiac output from thermodilution for calibration of esCCOTM [16] but still reported relatively large 95% prediction intervals of −2.13 to 2.39 l/min. The clinical benefit of this approach remains to be clarified, since the invasive techniques for catheter placement cannot be eliminated. Several other studies used invasive arterial blood pressure monitoring for calibration of esCCOTM [16–18, 22, 23]. Therefore, the method cannot be considered noninvasive in these studies. Our analysis of CO_esCCO_ revealed a percentage error of 47% compared with CO_TPTD_, which above the maximum is considered to be clinically acceptable (considered to be >30% [14]).

Smetkin et al. [24] recently compared esCCOTM with transpulmonary thermodilution by PiCCOTM in patients during and after off-pump coronary artery bypass grafting (OPCAB). Although OPCAB might not reflect a mixed ICU population, as this population contained no high output failures, the study reported limited agreement between the techniques with a bias of 0.9 l/min, limits of agreement of −0.9 l/min and +3.7 l/min, and a percentage error of 57%.

Our second goal was to assess the trending ability of esCCOTM. The detection of changes in cardiac output is a key aim of CO assessment, especially in patients with sepsis, for the assessment of volume responsiveness. The trending ability of esCCOTM has been tested in several studies, showing clinically acceptable results in stable patients during elective surgery [17, 18, 22], whereas the trending ability of esCCOTM in ICU patients was reported to be unreliable [19, 25, 26]. In our study, esCCOTM failed to detected changes in cardiac output from baseline at a clinically acceptable rate. Only a weak correlation between ΔCO_esCCO_ and ΔCO_TPTD_ was found (*r* = 0.442). An adjusted *r*^2^ of 0.195 shows that only around 20% of the variability estimated by CO_esCCO_ can be explained by changes observed by CO_TPTD_. We further found that esCCOTM underestimates changes in CO by a factor of nearly three. The concordance rate was 74%, meaning that in 74% of patients, both the methods registered changes in the same direction. This is, however, too low, since only rates ≥90% are considered sufficient to guide the therapy [15].

Our third aim was to assess the esCCOTM method in patients with different medical conditions. Frequent indications for monitoring CO include sepsis and polytrauma. Differences in pathophysiology potentially affect measurements of the pulse-wave transit time method and reduce its accuracy. In this study, the esCCOTM method did not yield acceptable agreement and precision (Table 2) in any of the subgroups (sepsis, polytrauma, and nonsepsis/nonpolytrauma). Further, in each subgroup, the TPTD and esCCOTM trends in cardiac output did not correlate significantly. Further, in each subgroup, trends in cardiac output did not correlate significantly between TPTD and esCCOTM. Several studies have reported a negative correlation between bias and systemic vascular resistance (SVR) [18, 19, 26] and questioned its usefulness in situations with a variable systemic vascular resistance. On the other hand, in settings with stable SVR, esCCOTM does not offer any benefit to mean arterial pressure readings [25].

Our study has several limitations. First, as a reference method, our “gold standard,” was the TPTD and pulse contour analysis, although both have their limitations. Nevertheless, TPTD is accurate [27] and can detect changes in CO comparable to a PAC [28, 29]. Second, we did not measure SVR or the use of vasoactive drugs, which may have influenced esCCOTM estimates [16]. Third, we analyzed a small population that was heterogenous in terms of clinical conditions and comorbidities. This makes it hard to identify the causes of the poor agreement and trending ability. Furthermore, the small sample size and related low analytic power precludes identification of potential subgroups, in which esCCOTM would yield clinically acceptable CO estimates. Future studies should focus on the strengths and weaknesses of esCCOTM in various settings to find a potential field of application in the setting of critical care.

## 5. Conclusion

In a mixed population of critically ill patients, the estimated continuous CO using the noninvasive method of pulse-wave transit time has low accuracy, low precision, and poor trending ability as compared with transpulmonary thermodilution and the pulse contour analysis. An acceptable agreement and trending ability could not be shown in patients with sepsis, polytrauma, or other conditions.

## Figures and Tables

**Table 1:** Characteristics of patients included.

	Patients with				Total
	Sepsis	Polytrauma	No sepsis/no polytrauma	Sepsis and polytrauma	
Count (number of patients), *n*	13	6	18	1	38
Male, *n* (%)	7 (54)	6 (100)	12 (67)	1 (100)	26 (68)
Age, years (median) and (IQR)	62 (55–73)	60 (51–69)	61 (47–72)	82 (82-82)	62 (50–72)
Weight, kg (median) and (IQR)	80 (75–90)	81 (71–88)	74 (58–83)	72 (72-72)	77 (70–84)
Height, cm (median) and (IQR)	172 (160–176)	180 (178–186)	176 (165–180)	176 (176-176)	176 (168–180)
SAPS II (median) and (IQR)	42 (37–56)	41 (26–55)	45 (34–50)	65 (65-65)	45 (34–54)
Heart rate, beats/min (median) and (IQR)	94 (85–110)	84.5 (70–99)	85 (76–95)	114 (114-114)	90 (79–101)
Respiratory rate, breaths/min (median (IQR))	18 (18–22)	16 (15–23)	18 (15–22)	20 (20-20)	18 (16–22)
Oxygen saturation, (%) (median (IQR))	98 (95–99)	100 (99-100)	98 (96–98)	95 (95-95)	98 (96–100)
Mean arterial pressure, mmHg (median (IQR))	70 (65–75)	77 (70–86)	77 (70–85)	95 (95-95)	75 (70–85)
Systolic blood pressure, mmHg (median (IQR))	116 (105–140)	120 (110–125)	128 (113–140)	160 (160-160)	120 (110–140)
Diastolic blood pressure, mmHg (median (IQR))	55 (50–61)	55 (54–60)	60 (50–65)	60 (60-60)	57 (50–61)

*n*: number of patients; IQR: interquartile range.

**Table 2:** Subgroups are listed in columns, and parameters are provided from correlation, the Bland–Altman analysis, and the trending analysis.

	Patients with sepsis	Patients with polytrauma	Patients with no sepsis/no polytrauma
Count (number of patients) *n*	13	6	18
Correlation coefficient *r* (*p* value)	0.813 (0.001)	0.645 (0.166)	0.669 (0.002)
Bias, l/min	1.52	1.10	1.77
Lower limit of agreement, l/min	−2.08	−1.44	−1.81
Upper limit of agreement, l/min	+5.12	+3.63	+5.34
Percentage error, (%)	49	34	53
Correlation coefficient *r* of trends (*p* value)	0.353 (0.286)	0.470 (0.424)	0.317 (0.215)

*n*, number of patients.

## Data Availability

The data used to support this study is provided in the supplementary material.

## Abbreviations

CO: Cardiac output
esCCOTM: Estimated continuous cardiac output
TPTD: Transpulmonary thermodilution.

## Supplementary Materials

Supplementary Materials The supplement materials are the raw patient data including demographics, underlying problem, and CO assessments either by VISMO or PiCCO.
