# Supplementary material for: Continuous Estimation of Cardiac Output in Critical Care: A Noninvasive Method Based on Pulse Wave Transit Time Compared with Transpulmonary Thermodilution
Source: Crit Care Res Pract. 2020 Jul 20;2020:8956372. doi: 10.1155/2020/8956372 (PMC7387954; doi:10.1155/2020/8956372)
Supplement: Supplementary Materials — The supplement materials are the raw patient data including demographics, underlying problem, and CO assessments either by VISMO or PiCCO. [file 8956372.f1.pdf]

Number	Age (Year)	sex, M/F	weight (kg)	hight (cm)	Major problem (Sepsis (1), Polytrauma (2), Nor	Start Picco Monitoring (on ICU day)	Diagnonis: Heart (yes (1), no (0))	Lung (yes (1), no (0))	kidney (yes (1), no (0))	Liver (yes (1), no (0))	Neurology (yes (1), no (0))	skin (yes (1), no (0))	Picco-MAP (mmHg)	SAPS II	Examination: Heart (Normal (1), abnormal (2), missing (3))	Lungs (Normal (1), abnormal (2), missing (3))	Neurology (Normal (1), abnormal (2), missing (	skin (Normal (1), abnormal (2), missing (3))	heartrate/min	BD syst Picco (mmHg)	BD diast Picco (mmHg)	RespiRate/min	SpO2 (%)
1	51	f	58	165	3	7	0	0	1	1	0	0	65	24	2	1	2	2	95	95	60	12	97
2	78	f	75	152	1	3	1	1	1	0	0	0	75	77	2	2	1	1	96	108	42	15	95
3	56	m	90	180	1	9	1	0	1	0	0	0	70	37	2	2	2	1	94	116	55	22	100
4	59	m	83	176	3	1	1	1	1	0	1	0	80	30	0	1	0	1	85	114	58	22	97
5	67	f	53	160	3	15	0	1	1	0	1	0	95	49	1	1	2	1	100	150	80	32	96
6	51	m	88	180	2	3	0	0	0	0	0	0	90	62	1	2	3	2	75	140	86	16	100
7	36	m	58	174	3	2	0	0	1	0	0	0	60	13	1	1	1	1	80	130	60	12	98
8	82	m	72	176	4	3	0	1	0	0	0	0	95	65	1	1	1	1	114	160	60	20	95
9	69	m	80	178	2	5	0	1	0	0	0	0	80	47	1	2	1	2	60	105	51	15	100
10	44	f	55	172	3	4	0	0	0	0	1	0	97	40	2	1	3	1	90	126	76	22	100
11	82	f	100	155	3	3	1	0	1	0	0	0	70	50	1	2	1	1	85	105	50	22	94
12	69	m	84	168	3	1	1	1	0	0	1	0	94	42	1	1	1	1	79	154	61	18	98
13	71	f	51	158	1	4	1	0	1	0	0	0	72	61	1	1	1	1	87	103	60	18	98
14	85	m	82	171	1	2	1	0	1	0	0	0	95	42	1	1	1	1	90	147	61	15	94
15	72	m	79	175	3	16	1	1	0	0	0	0	70	30	1	2	1	1	70	140	60	14	98
16	69	m	90	186	3	13	1	1	0	0	1	0	85	31	1	1	2	2	80	140	80	18	99
17	50	m	110	195	1	12	0	0	0	0	0	1	80	32	1	1	1	2	110	140	80	20	100
18	81	m	90	160	1	15	1	1	0	0	0	0	65	38	1	2	1	1	102	105	45	20	99
19	43	m	75	186	3	5	0	0	0	0	1	0	100	54	1	1	2	1	110	150	80	28	95
20	71	f	68	160	3	14	0	0	1	0	0	1	60	48	1	1	1	2	90	130	50	20	98
21	63	f	78	174	1	5	0	1	1	1	0	0	85	61	1	2	1	1	110	146	61	18	95
22	73	f	70	157	1	4	1	0	1	0	0	0	65	24	2	1	1	2	120	90	50	18	98
23	78	m	70	185	3	15	0	1	0	0	1	1	70	52	1	1	2	2	76	130	55	12	99
24	47	m	81	186	2	1	0	0	0	0	0	0	74	55	1	2	2	1	70	120	54	15	100
25	58	m	90	190	2	7	0	1	0	0	1	0	70	25	1	1	1	1	104	110	55	23	99
26	47	m	81	177	3	7	1	1	1	1	0	1	70	34	1	1	1	2	56	110	50	16	97
27	63	f	40	150	3	3	1	0	1	0	0	0	75	60	1	2	1	1	115	133	42	26	95
28	81	m	71	180	2	4	1	0	0	0	0	0	86	26	1	1	2	1	94	125	60	16	100
29	72	m	72	176	3	1	0	0	0	0	1	0	85	38	1	1	2	1	76	140	50	15	95
30	32	m	150	170	1	1	0	0	0	1	0	0	93	17	1	2	1	1	99	126	74	15	95
31	62	m	80	175	1	1	0	0	0	0	0	0	60	41	2	1	2	1	78	130	50	18	95
32	55	m	75	172	1	14	0	0	1	1	0	0	75	33	1	1	2	1	114	116	56	27	100
33	62	m	70	170	2	9	1	0	0	0	0	0	70	35	1	1	2	1	99	119	55	24	96
34	59	f	80	170	1	6	0	1	1	0	0	0	70	56	1	1	1	1	85	143	73	29	96
35	31	m	94	194	1	1	1	0	0	1	1	0	61	50	2	2	2	2	84	118	49	24	99
36	40	m	105	196	3	3	0	0	0	1	0	0	73	49	1	1	1	1	74	106	50	17	100
37	72	m	100	188	3	2	1	0	1	0	0	0	81	85	2	1	1	1	80	120	65	17	97
38	22	f	67	176	1	2	0	1	1	1	0	0	70	53	1	2	2	1	79	96	54	16	96
39	50	m	72	176	3	1	0	0	0	0	1	0	80	47	1	1	2	1	95	120	80	18	98
40	53	m	75	180	3	5	0	1	1	0	0	1	79	36	1	2	1	2	101	113	61	16	100

Number	PM1	PM2	PM3	PM4	PM5	PM6	VM1	VM2	VM3	VM4	VM5	VM6
1	6.4	7.2	7.34	6.82	6.51	7.64	4.86	5.09	4	2.84	4.11	3.52
2	3.89	4.58	6.03	4.91	4.73		6.47	5.89	7.01	5.99	6.11	
3	11.4	11.2	12.3	12.7	12.2	11	7.19	7.03	7.27	6.88	6.6	6.62
4	9.44	9.85	8.16	8.1	9.87	9.57	7.1	6.6	5.45	6.07	6.05	6.63
5	4.88	5.24	4.92	5.13	4.37	3.82	4.74	6.19	7.69	8.23	5.95	4.59
6	8.12	7.79	8.12	7.66	7.42	8.9	7.71	7.69	8.03	7.84	7.5	7.74
7	6.06	6.39	7.37	8.2	7.1	7.5	5.19	5.19	5.3	5.76	5.33	5.6
8	11	11.1	7.46	7.81	6.51	6.32	8.32	8.14	9.9	8.59	6.69	6.12
9	7.5	7.7	7.8	8.8	8.45	7.85	5	5	4.95	6.29	6.25	5.41
10	6.27	6.84	7.54	7.19	7.23	7.81	8.02	7.99	8.06	7.79	7.52	8.24
11	3	3.5	3.4	3.05	2.28	2.26	4.69	4.47	4.2	3.75	4.02	3.3
12	7.68	7.23	7.06	5.56	6.33	6.91	6.23	6.03	6.3	6.58	7.45	8.27
13	4.17	4.26	3.39	3.46	3.39	4.19	3.07	3.68	2.35	2.7	2.2	1.3
14	7.35	6.98	5.9	5.5	6.8	7.8	6.01	7.07	7.35	3.8	7.5	7.17
15	6.28	6.19	5.3	6.32	6.72	7.22	5.04	4.49	4.22	5.25	5.51	5.37
16												
17	13.7	13.4	13.4	8.84	13.6	10.4	12.91	12.05	12.78	13.18	13.05	11.79
18	5.15	7.38	7.9	6.89	6.11	5.67	5.3	4.69	5.27	4.3		
19	13	12.6	13.5	11.9	10.5	10.7	9.76	9.12	9.74	8.8	7.55	7.62
20	6.15	6.81	5.95	5.56	5.7	5.35	1.85	5.73	4.5	4.43	5.35	6.2
21	9.17	8.99	9.27	8.12	8.89	8.86	5.86	5.99	3.8	5.18	5.31	4.84
22	3.04	2.9	2.88	2.5	2.89	3.14	3.8	3.8	4.32	4.58	4.58	4.5
23	6.47	6.41	5.78	6.42	6.35	8.33	4.17	5.05	5.19	6.02	6.08	6.14
24	7.18	7.34	7.49	7.43	7.5	8.2	6.28	7.11	6.23	5.91	6.5	6.3
25	7.72	11.4					9.1	9.22				
26	6.7	7.15					5.18	4.22	4.35			6.33
27	8.6	8.56	7.29	7.22	5	7.5	3.95	4.99	4.06	2.34	2.45	2.61
28	5.06	5.84	5.47	5.76	6.2	5.83	5.47	5.66	5.99	5.61	6.66	6.52
29	7.4	7.64	7.24	6.85	8.25	7.38	6.48	6.37	6.13	6.47	7.38	6.93
30			11.2	11.4	11.2	11.2	9.85	10.13	10.66	10.27	10.18	10.41
31	6.74	6.92	6.61	6.03	6.35	7.6	6.35	6.46	6.39	5.94	6.5	6.37
32	8.7	9.42	9.16	9.87	10	10.4	7.38	6.39	6.95	8.01	7.05	7.84
33	9.97	9.31	8.42	8.75	8.49	7.85	6.7	6.85	7.11	6.99	9.06	6.04
34	9.22	9.96	9.75	9.17	7.27	7.94	7.72	8.04	8.09	7.54	9.87	6.99
35	10.6	10.5	11.4	11.7	11.2	12.1	8.08	7.84	8.77	7.77	7.21	7.23
36	11.2	11.3	11.3	11.8	12.1	10.7	7.75	7.96	7.99	7.93	9.06	7.79
37	5.92	6.16	6.44	6.66	6.58	6.31	6.67	6.59	7.22	6.96	6.71	6.88
38	10.2	10.2	10.2	10.9	10.6	10.1	5.71	5.7	6.89	6.4	6.58	6.17
39	12	10.9	11.5	11.4	12.1	12.3	7.32	7.38	7.67	8.13	8.09	7.9
40	9.21	10.8	11.2	11.2	11	11.3	5.34	5.82	6.19	4.77	5.15	5.15

Variable:

**name:**	**Method**	**investigator**	**time point**
PM1	Cardiac output estimate by PiCCO™	1	baseline
PM2	Cardiac output estimate by PiCCO™	2	baseline
PM3	Cardiac output estimate by PiCCO™	1	2h post baseline
PM4	Cardiac output estimate by PiCCO™	1	4h post baseline
PM5	Cardiac output estimate by PiCCO™	1	6h post baseline
PM6	Cardiac output estimate by PiCCO™	1	8h post baseline
VM1	Cardiac output estimate by Vismo™	1	baseline
VM2	Cardiac output estimate by Vismo™	2	baseline
VM3	Cardiac output estimate by Vismo™	1	2h post baseline
VM4	Cardiac output estimate by Vismo™	1	4h post baseline
VM5	Cardiac output estimate by Vismo™	1	6h post baseline
VM6	Cardiac output estimate by Vismo™	1	8h post baseline
